# Clinical and genetic features of Charcot‐Marie‐Tooth disease 2F and hereditary motor neuropathy 2B in Japan

**DOI:** 10.1111/jns.12252

**Published:** 2018-02-14

**Authors:** Hajime Tanabe, Yujiro Higuchi, Jun‐Hui Yuan, Akihiro Hashiguchi, Akiko Yoshimura, Satoshi Ishihara, Satoshi Nozuma, Yuji Okamoto, Eiji Matsuura, Hiroyuki Ishiura, Jun Mitsui, Ryotaro Takashima, Norito Kokubun, Kengo Maeda, Yuri Asano, Yoko Sunami, Yu Kono, Yasunori Ishigaki, Shosaburo Yanamoto, Jiro Fukae, Hiroshi Kida, Mitsuya Morita, Shoji Tsuji, Hiroshi Takashima

**Affiliations:** ^1^ Department of Neurology and Geriatrics Kagoshima University, Graduate School of Medical and Dental Sciences Kagoshima Japan; ^2^ Department of Cardiovascular Medicine, Nephrology and Neurology, Graduate School of Medicine University of the Ryukyus Okinawa Japan; ^3^ Department of Neurology, Graduate School of Medicine The University of Tokyo Tokyo Japan; ^4^ Department of Neurology Dokkyo Medical University Tochigi Japan; ^5^ Department of Neurology National Hospital Organization Higashi‐ohmi General Medical Center Shiga Japan; ^6^ Department of Neurology Tokyo Metropolitan Neurological Hospital Tokyo Japan; ^7^ Department of Neurology The Jikei University School of Medicine Tokyo Japan; ^8^ Department of Neurology Coral Clinic Tokyo Japan; ^9^ Department of Neurology Fukuoka University School of Medicine Fukuoka Japan; ^10^ Division of Respirology, Neurology, and Rheumatology, Department of Medicine Kurume University School of Medicine Fukuoka Japan; ^11^ Division of Neurology Jichi Medical University Tochigi Japan

**Keywords:** abnormal glucose metabolism, Charcot‐Marie‐Tooth disease 2F, distal hereditary motor neuropathy 2B, male predominance, next‐generation sequencing

## Abstract

Mutations in small heat shock protein beta‐1 (HspB1) have been linked to Charcot‐Marie‐Tooth (CMT) disease type 2F and distal hereditary motor neuropathy type 2B. Only four cases with HSPB1 mutations have been reported to date in Japan. In this study between April 2007 and October 2014, we conducted gene panel sequencing in a case series of 1,030 patients with inherited peripheral neuropathies (IPNs) using DNA microarray, targeted resequencing, and whole‐exome sequencing. We identified HSPB1 variants in 1.3% (13 of 1,030) of the patients with IPNs, who exhibited a male predominance. Based on neurological and electrophysiological findings, seven patients were diagnosed with CMT disease type 2F, whereas the remaining six patients were diagnosed with distal hereditary motor neuropathy type 2B. P39L, R127W, S135C, R140G, K141Q, T151I, and P182A mutations identified in 12 patients were described previously, whereas a novel K123* variant with unknown significance was found in 1 patient. Diabetes and impaired glucose tolerance were detected in 6 of the 13 patients. Our findings suggest that HSPB1 mutations result in two phenotypes of inherited neuropathies and extend the phenotypic spectrum of HSPB1‐related disorders.

## Introduction

Charcot‐Marie‐Tooth (CMT) disease is a common, clinically and genetically heterogeneous group of hereditary neuropathies. To date, more than 80 CMT disease‐causing genes have been identified. Another inherited peripheral neuropathy (IPN), distal hereditary motor neuropathy (HMN), resembles CMT but is a pure motor neuron disease with no or mild sensory nervous system involvement.

The *HSPB1* gene codes for heat shock protein beta‐1 (HspB1, also called heat shock protein 27), which is a member of the small heat shock protein family comprising a highly conserved α‐crystallin domain. HspB1 acts as a chaperone by binding misfolded or denatured proteins and preventing them from forming toxic aggregates *(Dierick et al.,* 2005 *; Arrigo,* 2007 *)*. Mutations in *HSPB1* were shown to be associated with CMT type 2F (CMT2F, OMIM 602195) with autosomal dominant or recessive inheritance and HMN type 2B (HMN2B, OMIM 608634) with minimal sensory involvement. Since 2004, more than 40 families with CMT2F/HMN2B due to missense and rarely nonsense or frameshift mutations in *HSPB1* have been reported *(Evgrafov et al.,* 2004 *)*.

An Italian study reported the frequencies of *HSPB1* mutations in HMN and CMT2 as 8% and 4%, respectively *(Capponi et al.,* 2011 *)*. However, in Japan, only four sporadic HMN2B cases due to *HSPB1* mutations have been reported thus far, and the clinical features remain poorly defined. Using DNA microarray and next‐generation sequencing (NGS) technologies, we identified *HSPB1* mutations in a large case series of Japanese patients and investigated the underlying clinical characteristics of CMT2F/HMN2B, including sex differences and abnormal glucose metabolism.

## Materials and Methods

### Patients

In this study, 1,030 patients with IPNs were enrolled. Clinical information of the patients was provided by nationwide medical facilities in Japan. The diagnostic criteria for CMT2 included progressive sensory and motor symptoms, signs of weakness and wasting, and conserved median motor nerve conduction velocities (>38 m/s) with reduced compound muscle action potential amplitudes. The diagnosis of HMN was based on distal lower and/or upper limb weakness and wasting, reduced reflexes, and pure motor axonal neuropathy confirmed by nerve conduction studies.

Genomic DNA was extracted from peripheral blood leukocytes of patients using the Gentra Puregene Blood Kit (Qiagen, Dusseldorf, Germany). Study protocols were reviewed and approved by the institutional review board of Kagoshima University. All patients and their family members who were referred to this study provided written informed consent.

Duplication/deletion mutation of *PMP22* was preexcluded in all clinically suspected demyelinating CMT cases using fluorescence *in situ* hybridization or multiplex ligation‐dependent probe amplification. In April 2007, we started mutation screening in patients with suspected CMT, hereditary neuropathy with liability to pressure palsies, or hereditary motor neuropathy who were referred to our laboratory.

### DNA resequencing microarrays

Between April 2007 and April 2012, a purpose‐built GeneChip^®^ CustomSeq^®^ Resequencing Array (Affymetrix, Santa Clara, CA, USA), which was used for screening 28 CMT and HMN disease‐causing genes, was initially utilized following the protocol described previously *(Zhao et al.,* 2012 *)*.

### Targeted resequencing using Illumina Miseq

Starting in May 2012, a targeted resequencing system using the Illumina Miseq platform (Illumina, San Diego, CA, USA) was introduced for mutation screening. This system targeted a panel of 60 disease‐causing or candidate genes of IPNs, and the specific design and workflow were illustrated previously *(Maeda et al.,* 2014 *)*.

### Whole‐exome sequencing

Among mutation‐negative cases identified by the previous screening, which was performed prior to May 2013, 398 cases that were highly suspicious for CMT were analyzed by whole‐exome sequencing using HiSeq 2000 (Illumina). Sequences were aligned to the human reference genome (NCBI37/hg19) using the Burrows–Wheeler Aligner. Variant calling was performed using SAMtools and was annotated using in‐house scripts.

### Variant validation and interpretation

All *HSPB1* variants were verified against the 1,000 Genome (http://browser.1000genomes.org/index.html), ExAC (http://exac.broadinstitute.org/), and Human Genetic Variation (http://www.genome.med.kyoto-u.ac.jp/SnpDB/) databases. Next, Sanger sequencing was used to validate the suspected variants, and a segregation analysis was performed whenever possible. Variants were interpreted according to the American College of Medical Genetics and Genomics/Association for Molecular Pathology (ACMG/AMP) guidelines published in 2015 *(Richards et al.,* 2015 *)*.

## Results

In this series, 13 unrelated patients harboring *HSPB1* variants were identified. Among these, 10 patients had inherited variants with an autosomal dominant pattern, whereas 3 patients had a sporadic pattern. Clinical diagnosis was CMT2F in seven patients and HMN2B in six patients.

**Figure 1:**
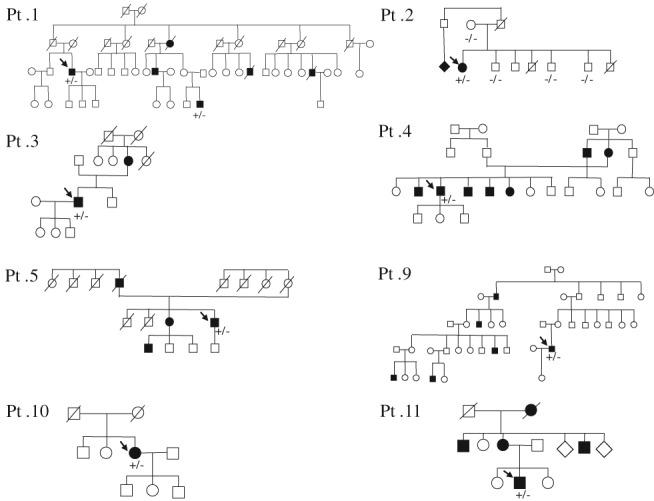
Pedigrees of the families with HSPB1 mutations. The probands are indicated by black arrows. +/− = heterozygous, −/− = homozygous normal for the HSPB1 mutation.

**Figure 2:**
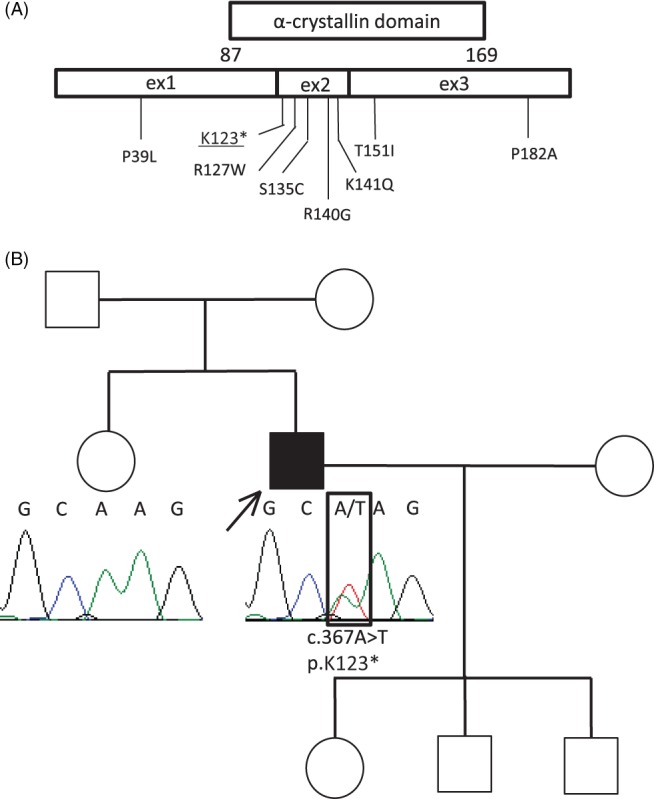
(A) HSPB1 variants identified in this study. The novel variant with unknown significance is underlined. (B) Family tree and sequencing chromatogram of patient 13. Heterozygous novel nonsense variant c.367A>T (K123*) was detected in the proband but was absent in the unaffected sister

Clinical, genetic, and electrophysiological features of all patients with *HSPB1* variants are summarized in Tables 1 and 2. The family trees of the patients were shown Figures 1, 2B and 4A. The following seven known heterozygous mutations were identified in 12 patients: T151I, P39L, R140G, R127W, S135C, K141Q, and P182A (Fig. 2A). The T151I mutation, which was found in four unrelated patients, was determined to be a mutation hot spot.

**Table 1:** Clinical features of the patients.

							Upper limbs		Lower limbs						
Pt	Mutation	Amino acid change	Inheritance	CMT2/HMN2B	Age/sex (y.o)	Onset (y.o)	DTR† (rt/lt)	Atrophy (rt/lt)	MMT‡ (TA)	DTR	Atrophy (rt/lt)	Sensory disturbance	Other clinical findings	DM/IGT	HbA1c§,¶
1**	c.421A>C	K141Q	D	HMN2B	69/M	68	±/±	−/−	4/4	±/±	+/+	No	Mild CK elevation (488 IU/l)	No	N.D
2	c.544C>G	P182A	D	CMT2F	49/F	19	+/+	+/+	4/4	±/±	+/+	Dysesthesia		No	N.D
3	c.116C>T	P39L	D	CMT2F	61/M	57	+/+	−/−	2/2	−/−	+/+	+		DM	8.5% (N.D)
4	c.116C>T	P39L	D	CMT2F	75/M	57	+/+	+/+	0/0	−/−	+/+	No	Mild CK elevation (281 IU/l)	DM	6.8% (NGSP)
5	c.379C>T	R127W	D	HMN2B	58/M	55	+/+	N.D	4/4	±/±	N.D	No		IGT	6.9% (NGSP)
6	c.418C>G	R140G	D	CMT2F	70/M	65	±/±	+/+	3/3	−/−	+/+	No	Mild CK elevation (426 IU/l)	IGT	6.7% (N.D)
7	c.418C>G	R140G	D	HMN2B	68/M	50	+/+	−/−	2/2	+/+	+/+	No	Dysphagia, fasciculation.	No	N.D
8	c.404C>G	S135C	S	CMT2F	64/M	59	±/±	−/−	0/0	−/−	+/+	+	Mild CK elevation (912 IU/l)	DM	7.3% (JDS)
9	c.452C>T	T151I	D	HMN2B	63/M	61	N.D	N.D	2/2	−/−	N.D	No		No	N.D
10	c.452C>T	T151I	S††	HMN2B	61/F	52	+/+	N.D	5/0	+/+	N.D	No		DM	6.1% (N.D)
11	c.452C>T	T151I	D	CMT2F	62/M	51	N.D	N.D	1/1	±/±	N.D	No		No	N.D
12	c.452C>T	T151I	D	HMN2B	45/M	40	+/+	−/−	1/1	−/−	+/+	No	PH of hyperglycemia Mild CK elevation (318 IU/l)	No	5.8% (NGSP)
13	c.367A>T	K123*	S	CMT2F	61/M	51	+/+	−/−	4/4	−/−	+/+	+	Mild CK elevation (300–400 IU/l)	No	N.D

ATR, Achilles tendon reflex; CK, Creatine kinase; D, dominant; DM, diabetes mellitus; F, female; IGT, impaired glucose tolerance; JDS, Japan Diabetes Society; lt, left; M, male; MMT, manual muscle test; N.D, no data; NGSP, National Glycohemoglobin Standardization Program; OPLL, ossification of posterior longitudinal ligament; PH, past history; PTR, Patella tendon reflex; rt., right; S, sporadic; TA, tibialis anterior; y.o, years old.

† DTR: +, normal; ±, decreased; −, absent.

‡ MMT: 5, normal; 4, good; 3, fair; 2, poor; 1, trace.

§ The highest HbA1c value within the data provided by the primary physician.

¶ Cut‐off value of HbA1c for the diagnosis of DM is 6.1% in JDS criteria and 6.5% in NGSP criteria.

** Data of the father and brother is not available.

†† *Maeda et al. (* 2014 *)*.

**Table 2:** Electrophysiological findings of the patients.

	Median nerve				Ulnar nerve				Tibial nerve			
	Motor		Sensory		Motor		Sensory		Motor		Sural nerve	
Pt	Amp (mV)	CV (m/s)	Amp (μV)	CV (m/s)	Amp (mV)	CV (m/s)	Amp (μV)	CV (m/s)	Amp (mV)	CV (m/s)	Amp (μV)	CV (m/s)
1	7.3	52	24.0	45	4.7	61	24.5	54	1.1	33	4.2	42
2	4.9	50	34.0	58	2.8	56	13.0	50	0.1	N.R	4.0	46
3	15.1	45	24.7	53	15.3	46	15.9	47	0.1	34	2.9	43
4	9.9	56	11.0	45	N.D		N.D		N.R		N.R	
5	10.2	38	31.3	9	18.3	63	32.9	53	N.R		2.3	50
6	N.D	W.N.L	N.D	W.N.L	N.D		N.D		0.3	W.N.L	N.D	
7	18.9	55	9.0	48	24.2	58	11.0	48	N.D		N.D	
8	7.0	58	15.7	55	4.7	52	N.D		0.4	43	5.3	15
9	11.0	63	32.9	52	12.3	58	23.5	53	0.2	44	7.4	47
10	N.D	≧38	N.D		N.D	≧38	N.D		N.R		N.D	55
11	4.6	64	19.3	56	8.6	61	18.0	57	N.R		2.8	41
12	8.2	58	13.0	52	8.3	66	11.0	56	N.R		8.0	39
13	14.4	41	7.9	41	14.9	45	11.0	40	N.R		3.1	34

Amp, amplitude; CV, conduction velocity; N.D, no data; N.R, not recorded; W.N.L, within normal limit.

A novel heterozygous nonsense c.367A>T (K123*) variant was detected in a 61‐year‐old patient with sporadic CMT2F (patient 13) with asymmetrical weakness and mild sensory disturbance in the distal legs.

**Figure 3:**
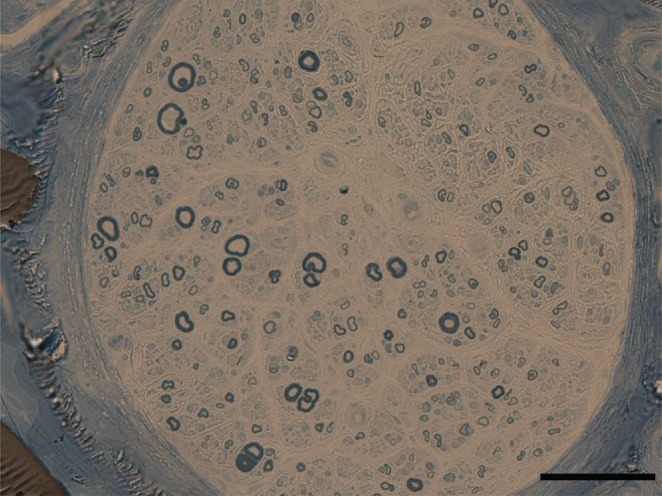
Sural nerve pathology of patient 4. The density of large myelinated fibers is markedly decreased. No onion bulb formation is observed (toluidine blue, ×200. Scale bar, 50 μm)

Among the 13 patients in our study, the mean age of onset was 53 ± 12 years, which was slightly higher than that reported previously *(Tang et al.,* 2005 *)*. No sensory abnormalities were identified in the upper limbs of any of the patients using nerve conduction studies. The difference in the mean age of onset (51 vs. 54 years) was not different between the CMT2F and HMN2B groups. However, disease duration for patients with CMT2F (12 ± 9 years) was longer than that of patients with HMN2B (6 ± 6 years). Pathological analysis of a sural nerve biopsy performed in patient 4 showed a reduction in large myelinated fibers (3,268/mm^2^) and axonal degeneration without onion bulb formation (Fig. 3).

**Figure 4:**
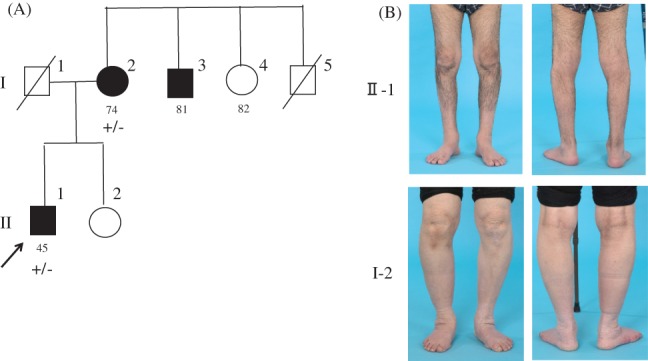
(A) Pedigree of patient 12. The proband is indicated by a black arrow. The numeric under each family member indicates age (years old). (B) Clinical pictures of the bowlegged patient 12 (II‐1) and his mother (I‐2). Atrophy of the distal lower extremities is evident in patient 12 but is inconspicuous in the mother. Pes cavus is not observed. +/− = heterozygous for the HSPB1 mutation.

We noticed a sex imbalance in our patients, with 11 males and 2 females (Table 1). In Family 12, although harboring the same T151I mutation, the male proband exhibited a much more severe clinical phenotype than his mother with slight foot weakness (Fig. 4B). To clarify the presence of a sex difference, we reviewed the literature for patients with IPNs caused by HSPB1 mutations, which revealed a comparable male predominance (67 males vs. 26 females, despite the comparable numbers of non‐affected male and female carriers (73 vs. 78, respectively; Table 3).

**Table 3:** Number of affected and non‐affected members in current and previous reports for each mutation.

		Male		Female	
Mutation	Author (year)	Affected (n)	Non‐affected (n) (carrier)	Affected (n)	Non‐affected (n) (carrier)
R127W	*Tang et al. (* 2005 *)*	8	13 (1)	2	16 (2)
	*Dierick (* 2008 *)*	4	0	3	0
	*Benedetti (* 2010 *)*	1	N.D	0	N.D
	Current report	3	7	1	1
T151I	*Dierick (* 2008 *)*	7	2	4	3
	*Nishibayashi (* 2007 *)*	2	N.D	2	N.D
	Current report	11	10	3	22 (1)
R140G	*Houlden (* 2008 *)*	5	7	0	4
	Current report	5	N.D	0	N.D
K141Q	*Ikeda et al. (* 2009 *)*	3	10	0	5
	Current report	5	12	1	18
S135C	*Benedetti (* 2010 *)*	0	N.D	2	N.D
	*Oberstadt (* 2016 *)*	2	1	3	0
	Current report	1	N.D	0	N.D
P182A	Current report	0	6	1	0
P39L	*Houlden (* 2008 *)*	1	0	1	0
	*Capponi et al. (* 2011 *)*	2	0	0	1
	Current report	6	5	3	7
K123*	Current report	1	0	0	1
Total^a^		67	73	26	78

n, number; N.D, no data.

a Total number of pedigrees in all mutations.

In our series, diabetes mellitus (DM) was found in patients 3, 4, 8, and 10 with disease durations of 6, >2, unknown, and 6 years, respectively. Impaired glucose tolerance (IGT) was identified in patients 5 and 6. Moreover, patient 12 reported a history of hyperglycemia. However, no notable clinical or electrophysiological difference was found between patients with and without DM/IGT.

## Discussion

This study identified *HSPB1* mutations in 13 Japanese CMT2F/HMN2B pedigrees and summarized their clinical and genetic characteristics. Overall, the clinical features of the current case series resemble those previously reported. We found *HSPB1* mutations in 1.3% (13 of 1,030) of the patients clinically diagnosed with IPNs in our series, which was comparable with the frequency of the *HSPB1* mutations reported in the United States (0.3% in IPNs), Taiwan (0.4% in CMT), and Korea (0.6% in CMT and HMN) *(Capponi et al.,* 2011 *; DiVincenzo et al.,* 2014 *; Lin et al.,* 2011 *)*.

HspB1 is a 205‐amino acid protein which contains a signature α‐crystallin domain at residues Glu87‐Pro168 flanked by the N‐ and C‐termini. The mutant HspB1 can form intracellular aggregates, inhibit cell division, or disrupt the neurofilament network *(Ackerley et al.,* 2006 *; Zhai et al.,* 2007 *)*. The α‐crystallin domain, which has an immunoglobulin‐like fold, mediates dimerization of individual protomers that subsequently assemble into larger oligomers *(Baranova et al.,* 2011 *)*. Using DNA microarrays and NGS, we identified seven previously described *HSPB1* mutations in 12 patients with CMT2F/HMN2B. Five of these mutations, P127W, S135C, R140G, K141Q, and T151I, are distributed in the center α‐crystallin domain and may disrupt the assembly of HspB1. The T151I mutation, which was identified in four patients, could be attributed to a founder ancestor due to their geographical clustering.

A heterozygous nonsense variant (K123*) was discovered in the current study. Nonsense mutations are not common among the known *HSPB1* mutations. As a reference, another heterozygous nonsense mutation, E175*, was previously reported *(Rossor et al.,* 2012 *)*. We interpret this K123* variant as unknown significance on the basis of following reasons: (1) absent in any of the global or Japanese database, including 1,000 Genomes, ExAC, Human Genetic Variation Database (HGVD), and integrative Japanese Genome Variation Database (iJGVD); (2) not detected in his unaffected sister; (3) positioned in the highly conserved α‐crystallin domain; (4) comparable clinical phenotype with other patients with *HSPB1* mutations; and (5) could escape from nonsense‐mediated mRNA decay, because of specific mechanism of the stress‐responsive genes *(Guo et al.,* 2009 *; Lykke‐Andersen and Jensen,* 2015 *)*.

The P182A mutation in patient 2, one of the three substitutions at residue P182 (others are P182L and P182S), was reported previously *(Evgrafov et al.,* 2004 *; Kijima et al.,* 2005 *; Rossor et al.,* 2017 *)*. Except for the prominent weakness of the distal upper extremities observed in the current patient, the neurological findings and the age of onset were comparable with the previous reports.

In the current study, we also performed a meta‐analysis to confirm the male predominance. The sex difference was prominent particularly in certain mutations such as R140G (10:0) and K141Q (8:1). Further, in family 12, we noted that the mother of the proband, although harboring T151I mutation, presented with relatively slight symptoms. A similar pattern was also observed in families 1 and 11; however, genetic analysis of other family members could not be conducted. These observations support the finding of an incomplete penetrance of *HSPB1* mutations, particularly in females. The significance of the sex difference in disease severity requires further investigations.

Mutant HspB1 affects the binding of nuclear factors (NFs) to the anterograde motor protein kinesin, thereby reducing the anterograde transport of NFs *(Holmgren et al.,* 2013 *)*. Two mutations in *HSPB1* (p.Gln190His and p.Al204Glyfs^*^6) were reported recently in patients with amyotrophic lateral sclerosis (ALS) phenotype, which suggested overlap of ALS and CMT2F/HMN2B *(Capponi et al.,* 2016 *)*. In the current study, patient 7 was suspected to have ALS because of dysphagia, fasciculation in left leg, and preserved deep tendon reflexes.

On the other hand, as with the findings in six of our patients (1.2 to 3.9 times of normal range), mild CK elevation was repetitively reported in patients with *HSPB1* mutations. Recently, a new phenotype of distal myopathy was described *(Lewis‐Smith et al.,* 2016 *)*, but in another report, no myogenic change was recognized in either needle electromyography or muscle biopsy *(Echaniz‐Laguna et al.,* 2017 *)*. In the present study, needle electromyography was recorded in four patients (patients 4, 6, 12, 13) with mild CK elevation, but none of them manifested with myogenic changes. Thus, the impairment of muscle membrane integrity due to denervation from axonal loss could be the likely reason for mild CK elevation in our study.

Impaired glucose metabolism, including DM and IGT, was verified in 6 of 13 patients (7 patients if patient 12 with a history of hyperglycemia is included). In half of these six patients, sensory disturbance was identified in the clinic or with nerve conduction studies, and it was difficult to clearly attribute these abnormalities to the CMT2F/HMN2B or DM diagnosis. Patients with *HSPB1* mutations were reported to be likely to have complications with glycemic abnormalities, as observed in a Japanese patient with the K141Q mutation *(Ikeda et al.,* 2009 *)*. Induction of heat shock proteins may combat insulin resistance *(McCarty,* 2006 *)*, and elevated serum HspB1 levels were also reported in patients with diabetic polyneuropathy *(Gruden et al.,* 2008 *)*. Therefore, it remains possible that the mutant HspB1 might act in concert with the hyperglycemic state to lower the onset threshold of DM or *vice versa*.

## References

[jns12252-bib-0001] Ackerley S , James PA , Kalli A , French S , Davies KE , Talbot K (2006). A mutation in the small heat‐shock protein HSPB1 leading to distal hereditary motor neuronopathy disrupts neurofilament assembly and the axonal transport of specific cellular cargoes. Hum Mol Genet 15:347–354. 16368711 10.1093/hmg/ddi452

[jns12252-bib-0002] Arrigo AP (2007). The cellular “networking” of mammalian Hsp27 and its functions in the control of protein folding, redox state and apoptosis. Adv Exp Med Biol 594:14–26. 17205671 10.1007/978-0-387-39975-1_2

[jns12252-bib-0003] Baranova EV , Weeks SD , Beelen S , Bukach OV , Gusev NB , Strelkov SV (2011). Three‐dimensional structure of alpha‐crystallin domain dimers of human small heat shock proteins HSPB1 and HSPB6. J Mol Biol 411:110–122. 21641913 10.1016/j.jmb.2011.05.024

[jns12252-bib-0024] Benedetti S , Previtali SC , Coviello S , Scarlato M , Cerri F , Di Pierri E , Piantoni L , Spiga I , Fazio R , Riva N , Natali Sora MG , Dacci P , Malaguti MC , Munerati E , Grimaldi LM , Marrosu MG , De Pellegrin M , Ferrari M , Comi G , Quattrini A , Bolino A (2010). Analyzing histopathological features of rare charcot‐marie‐tooth neuropathies to unravel their pathogenesis. Arch Neurol 67:1498–1505. 21149811 10.1001/archneurol.2010.303

[jns12252-bib-0004] Capponi S , Geroldi A , Fossa P , Grandis M , Ciotti P , Gulli R , Schenone A , Mandich P , Bellone E (2011). HSPB1 and HSPB8 in inherited neuropathies: study of an Italian cohort of dHMN and CMT2 patients. J Peripher Nerv Syst 16:287–294. 22176143 10.1111/j.1529-8027.2011.00361.x

[jns12252-bib-0005] Capponi S , Geuens T , Geroldi A , Origone P , Verdiani S , Cichero E , Adriaenssens E , De Winter V , Bandettini di Poggio M , Barberis M , Chiò A , Fossa P , Mandich P , Bellone E , Timmerman V (2016). Molecular chaperones in the pathogenesis of amyotrophic lateral sclerosis: the role of HSPB1. Hum Mutat 37:1202–1208. 27492805 10.1002/humu.23062 PMC5108433

[jns12252-bib-0006] Dierick I , Irobi J , De Jonghe P , Timmerman V (2005). Small heat shock proteins in inherited peripheral neuropathies. Ann Med 37:413–422. 16203614 10.1080/07853890500296410

[jns12252-bib-0030] Dierick I , Baets J , Irobi J , Jacobs A, De Vriendt E, Deconinck T, Merlini L, Van den Bergh P, Rasic VM, Robberecht W, Fischer D, Morales RJ, Mitrovic Z, Seeman P, Mazanec R, Kochanski A, Jordanova A, Auer‐Grumbach M, Helderman‐van den Enden AT, Wokke JH, Nelis E, De Jonghe P, Timmerman V (2008). Relative contribution of mutations in genes for autosomal dominant distal hereditary motor neuropathies: a genotype‐phenotype correlation study. Brain 131:1217–1227. 18325928 10.1093/brain/awn029

[jns12252-bib-0025] DiVincenzo C , Elzinga CD , Medeiros AC , Karbassi I , Jones JR , Evans MC , Braastad CD , Bishop CM , Jaremko M , Wang Z , Liaquat K , Hoffman CA , York MD , Batish SD , Lupski JR , Higgins JJ (2014). The allelic spectrum of Charcot‐Marie‐Tooth disease in over 17,000 individuals with neuropathy. Mol Genet Genomic Med 2:522–529. 25614874 10.1002/mgg3.106 PMC4303222

[jns12252-bib-0007] Echaniz‐Laguna A , Geuens T , Petiot P , Péréon Y , Adriaenssens E , Haidar M , Capponi S , Maisonobe T , Fournier E , Dubourg O , Degos B , Salachas F , Lenglet T , Eymard B , Delmont E , Pouget J , Juntas Morales R , Goizet C , Latour P , Timmerman V , Stojkovic T (2017). Axonal neuropathies due to mutations in small heat shock proteins: clinical, genetic, and functional insights into novel mutations. Hum Mutat 38:556–568. 28144995 10.1002/humu.23189

[jns12252-bib-0008] Evgrafov OV , Mersiyanova I , Irobi J , Van Den Bosch L , Dierick I , Leung CL , Schagina O , Verpoorten N , Van Impe K , Fedotov V , Dadali E , Auer‐Grumbach M , Windpassinger C , Wagner K , Mitrovic Z , Hilton‐Jones D , Talbot K , Martin JJ , Vasserman N , Tverskaya S , Polyakov A , Liem RK , Gettemans J , Robberecht W , De Jonghe P , Timmerman V (2004). Mutant small heat‐shock protein 27 causes axonal Charcot‐Marie‐Tooth disease and distal hereditary motor neuropathy. Nat Genet 36:602–606. 15122254 10.1038/ng1354

[jns12252-bib-0009] Gruden G , Bruno G , Chaturvedi N , Burt D , Schalkwijk C , Pinach S , Stehouwer CD , Witte DR , Fuller JH , Perin PC , EURODIAB Prospective Complications Study Group (2008). Serum heat shock protein 27 and diabetes complications in the EURODIAB prospective complications study: a novel circulating marker for diabetic neuropathy. Diabetes 57:1966–1970. 18390793 10.2337/db08-0009 PMC2453614

[jns12252-bib-0010] Guo K , Kang NX , Li Y , Sun L , Gan L , Cui FJ , Gao MD , Liu KY (2009). Regulation of HSP27 on NF‐kappaB pathway activation may be involved in metastatic hepatocellular carcinoma cells apoptosis. BMC Cancer 9:100. 19331697 10.1186/1471-2407-9-100 PMC2681475

[jns12252-bib-0011] Holmgren A , Bouhy D , De Winter V , Asselbergh B , Timmermans JP , Irobi J , Timmerman V (2013). Charcot‐Marie‐Tooth causing HSPB1 mutations increase Cdk5‐mediated phosphorylation of neurofilaments. Acta Neuropathol 126:93–108. 23728742 10.1007/s00401-013-1133-6 PMC3963106

[jns12252-bib-0026] Houlden H , Laura M , Wavrant‐De Vrièze F , Blake J , Wood N , Reilly MM (2008). Mutations in the HSP27 (HSPB1) gene cause dominant, recessive, and sporadic distal HMN/CMT type 2. Neurology 71:1660–1668. 18832141 10.1212/01.wnl.0000319696.14225.67

[jns12252-bib-0012] Ikeda Y , Abe A , Ishida C , Takahashi K , Hayasaka K , Yamada M (2009). A clinical phenotype of distal hereditary motor neuronopathy type II with a novel HSPB1 mutation. J Neurol Sci 277:9–12. 18952241 10.1016/j.jns.2008.09.031

[jns12252-bib-0013] Kijima K , Numakura C , Goto T , Takahashi T , Otagiri T , Umetsu K , Hayasaka K (2005). Small heat shock protein 27 mutation in a Japanese patient with distal hereditary motor neuropathy. J Hum Genet 50:473–476. 16155736 10.1007/s10038-005-0280-6

[jns12252-bib-0014] Lewis‐Smith DJ , Duff J , Pyle A , Griffin H , Polvikoski T , Birchall D , Horvath R , Chinnery PF (2016). Novel HSPB1 mutation causes both motor neuronopathy and distal myopathy. Neurol Genet 2:e110. 27830184 10.1212/NXG.0000000000000110 PMC5089436

[jns12252-bib-0027] Lin KP , Soong BW , Yang CC , Huang LW , Chang MH , Lee IH , Antonellis A , Lee YC (2011). The mutational spectrum in a cohort of Charcot‐Marie‐Tooth disease type 2 among the Han Chinese in Taiwan. PLoS One 6:e29393. 10.1371/journal.pone.0029393 PMC3242783 22206013

[jns12252-bib-0015] Lykke‐Andersen S , Jensen TH (2015). Nonsense‐mediated mRNA decay: an intricate machinery that shapes transcriptomes. Nat Rev Mol Cell Biol 16:665–677. 26397022 10.1038/nrm4063

[jns12252-bib-0016] Maeda K , Idehara R , Hashiguchi A , Takashima H (2014). A family with distal hereditary motor neuropathy and a K141Q mutation of small heat shock protein HSPB1. Intern Med 53:1655–1658. 25088881 10.2169/internalmedicine.53.2843

[jns12252-bib-0017] McCarty MF (2006). Induction of heat shock proteins may combat insulin resistance. Med Hypotheses 66:527–534. 16309849 10.1016/j.mehy.2004.08.033

[jns12252-bib-0028] Nishibayashi M , Kokubun N , Nakamura A , Hirata K , Yamamoto M , Sobue G (2007). Distal hereditary motor neuropathy type II with mutation in heat shock protein 27 gene. A case report. Rinsho Shinkeigaku 47:50–52. 17491338

[jns12252-bib-0029] Oberstadt M , Mitter D , Classen J , Baum P (2016). Late onset dHMN II caused by c.404C>G mutation in HSPB1 gene. J Peripher Nerv Syst 21:111–113. 26887567 10.1111/jns.12165

[jns12252-bib-0018] Richards S , Aziz N , Bale S , Bick D , Das S , Gastier‐Foster J , Grody WW , Hegde M , Lyon E , Spector E , Voelkerding K , Rehm HL , Committee ALQA (2015). Standards and guidelines for the interpretation of sequence variants: a joint consensus recommendation of the American College of Medical Genetics and Genomics and the Association for Molecular Pathology. Genet Med 17:405–424. 25741868 10.1038/gim.2015.30 PMC4544753

[jns12252-bib-0019] Rossor A , Davidson G , Blake J , Polke J , Murphy S , Houlden H , Innes A , Kalmar B , Greensmith L , Reilly M (2012). A novel p.Glu175X premature stop mutation in the C‐terminal end of HSP27 is a cause of CMT2. J Peripher Nerv Syst 17:201–205. 22734906 10.1111/j.1529-8027.2012.00400.x

[jns12252-bib-0020] Rossor AM , Morrow JM , Polke JM , Murphy SM , Houlden H , INC‐RDCRC , Laura M , Manji H , Blake J , Reilly MM (2017). Pilot phenotype and natural history study of hereditary neuropathies caused by mutations in the HSPB1 gene. Neuromuscul Disord 27:50–56. 27816334 10.1016/j.nmd.2016.10.001 PMC5260843

[jns12252-bib-0021] Tang B , Liu X , Zhao G , Luo W , Xia K , Pan Q , Cai F , Hu Z , Zhang C , Chen B , Zhang F , Shen L , Jiang H (2005). Mutation analysis of the small heat shock protein 27 gene in chinese patients with Charocot‐Marie‐tooth disease. Arch Neurol 62:1201–1207. 16087758 10.1001/archneur.62.8.1201

[jns12252-bib-0022] Zhai J , Lin H , Julien JP , Schlaepfer WW (2007). Disruption of neurofilament network with aggregation of light neurofilament protein: a common pathway leading to motor neuron degeneration due to Charcot‐Marie‐Tooth disease‐linked mutations in NFL and HSPB1. Hum Mol Genet 16:3103–3116. 17881652 10.1093/hmg/ddm272

[jns12252-bib-0023] Zhao Z , Hashiguchi A , Hu J , Sakiyama Y , Okamoto Y , Tokunaga S , Zhu L , Shen H , Takashima H (2012). Alanyl‐tRNA synthetase mutation in a family with dominant distal hereditary motor neuropathy. Neurology 78:1644–1649. 22573628 10.1212/WNL.0b013e3182574f8f PMC3359507

